# Detection and disclosure of workplace mental health challenges: an exploratory study from India

**DOI:** 10.1186/s12889-024-19422-9

**Published:** 2024-07-14

**Authors:** Ankur Poddar, Raina Chhajer

**Affiliations:** Indian Institute of Management Indore, Faculty Office J-223, Indore, India

**Keywords:** Workplace mental health challenges, Detection, Disclosure, Supportive organizational practices, India

## Abstract

Workplace mental health challenges have emerged as a significant concern post-pandemic. Despite this, the pervasive stigma surrounding mental illness leads to the concealment of symptoms and reluctance to seek professional help among employees. This study aims to explore the perception of different stakeholders towards the ‘Detection and disclosure’ of workplace mental health challenges in the Indian context. Fifteen semi-structured interviews were conducted with human resource professionals, counselors, and employees who had previously experienced mental health challenge(s). Thematic analysis was done to identify recurring themes and sub-themes. Three critical pathways were identified: *minimizing the inhibitory factors*, including lack of awareness, denial, low self-efficacy, stigma, and underestimating organizational capability; *maximizing the encouraging factors*, including psychological safety, perceived social support, and communicating success stories; and *implementing supportive organizational practices*, including generating awareness and literacy, build the organizational capability, strengthen the role of managers, leadership advocacy, policies, and processes. By fostering a culture of support and prioritizing employee well-being, organizations in India can create healthier and more resilient work environments, benefiting both individuals and the larger society.

**Supplementary Information:**

The online version contains supplementary material available at 10.1186/s12889-024-19422-9.

## Introduction

Workplace mental health challenges have emerged as a significant global concern, affecting individuals, organizations, and societies at large. With increasing awareness of mental health challenges, there has been a growing recognition of the impact of these challenges on employee well-being, organizational productivity, and overall economic prosperity [1–3]. The discourse surrounding workplace mental health has evolved, shedding light on the complexities of detection, disclosure, and management of mental health challenges in diverse organizational settings [4].

In recent years, the focus on workplace mental health has extended to the Indian context, where unique socio-cultural dynamics intersect with economic and organizational structures to shape the experiences of employees [5]. India, as a rapidly developing economy, grapples with the dual burden of traditional stigmas surrounding mental health and the modern-day pressures of a competitive corporate environment [6]. Understanding the intricacies of workplace mental health challenges in India requires a nuanced examination of cultural norms, organizational practices, and access to mental health resources [7, 8].

Detection and disclosure of workplace mental health challenges represent critical facets of addressing these issues effectively. Across diverse contexts, the ability to identify early signs of mental distress and create supportive environments for disclosure is pivotal in facilitating timely interventions and fostering a culture of well-being [9–11]. However, the challenges associated with detection and disclosure vary across different cultural and organizational contexts.

The detection of mental health challenges in the workplace involves identifying signs and symptoms of psychological distress among employees. Research suggests that fostering a supportive organizational culture, characterized by open communication and destigmatization of mental health issues, is essential for effective detection [12]. Supervisors and managers play a crucial role in recognizing behavioral changes, performance issues, and absenteeism that may indicate underlying mental health concerns [1]. Furthermore, the utilization of regular check-ins, anonymous surveys, and psychological screening tools has been proposed as effective strategies for detecting mental health challenges among employees [12].

In the Indian context, the detection of mental health issues is particularly challenging due to cultural stigmas and a lack of awareness about mental health. Traditional beliefs and social norms often discourage open discussions about mental health, leading to underreporting and inadequate support for affected employees [5]. Employers in India are increasingly recognizing the importance of mental health and are beginning to implement measures such as mental health awareness programs and employee assistance programs (EAPs) to better detect and address mental health issues [8].

Disclosure refers to the process by which employees communicate their mental health issues to their employers or colleagues. Literature emphasizes the importance of creating an environment of trust and psychological safety, wherein employees feel comfortable disclosing their mental health challenges without fear of negative consequences [13]. Organizational policies that protect employee confidentiality and prohibit discrimination based on mental health status are critical for facilitating disclosure [14]. Moreover, providing multiple channels for disclosure, such as confidential hotlines, HR representatives, or peer support networks, can enhance accessibility and encourage employees to seek help when needed.

In India, the stigma surrounding mental health often deters employees from disclosing their mental health challenges. A study highlights that fear of job loss, social ostracism, and lack of managerial support are significant barriers to disclosure in Indian workplaces [15]. To address these issues, Indian organizations are increasingly focusing on creating supportive environments and implementing policies that encourage disclosure while protecting employee confidentiality [16].

Detection and disclosure of workplace mental health challenges represent critical facets of addressing these issues effectively. Across diverse contexts, the ability to identify early signs of mental distress and create supportive environments for disclosure is pivotal in facilitating timely interventions and fostering a culture of well-being [9–11, 17]. However, the challenges associated with detection and disclosure vary across different cultural and organizational contexts.

The stigma surrounding mental illness often acts as a barrier to both detection and disclosure in the workplace [18–20]. Employees may fear judgment, discrimination, or professional repercussions if they disclose their mental health concerns, leading to underreporting and reluctance to seek help [21]. Moreover, the invisibility of mental health symptoms and the lack of awareness among employers and colleagues further complicate the detection process, resulting in missed opportunities for early intervention and support [22–24].

In the Indian context, cultural beliefs and societal norms significantly shape attitudes toward mental health, influencing detection and disclosure behaviors [25, 26]. The pervasive stigma surrounding mental illness often leads to the concealment of symptoms and reluctance to seek professional help, particularly in hierarchical work environments [5]. Additionally, the lack of mental health literacy among employers and employees further exacerbates the challenges of detection and disclosure, perpetuating misconceptions and hindering access to appropriate care [8, 27, 28].

Addressing workplace mental health challenges requires a multifaceted approach considering the interplay of individual, organizational, and societal factors. Organizations increasingly recognize the importance of creating supportive work environments prioritizing mental well-being and fostering open communication [1, 28, 29]. This entails implementing policies and programs to reduce stigma, providing mental health education and training, and offering accessible support services for employees [30–32]. Raising awareness, destigmatizing mental health discussions, and integrating mental health initiatives into broader public health agendas are critical in the Indian context [26, 33, 34]. By fostering a culture of support and prioritizing employee well-being, organizations in India can create healthier and more resilient work environments, benefiting both individuals and the larger society.

Detection of mental health challenges and mitigating the stigma of disclosing such challenges by employees is an emerging concept not yet extensively examined in the context of Indian organizations. Therefore, this exploratory study examines the perception of different stakeholders involved in addressing these challenges. This would enable the generation of new insights and theory development. We employed a qualitative research methodology for our study. Semi-structured interviews were thus conducted with different stakeholders, including HR professionals, counselors, and employees who had previously experienced mental health challenge(s).

## Method

This study is situated within the interpretivist paradigm. Interpretivism is selected because it aligns with our aim to understand the subjective experiences and perceptions of different stakeholders regarding workplace mental health challenges. This paradigm allows us to explore the nuanced and contextualized meanings that individuals attach to their experiences and actions. Our ontological stance is constructivist, which posits that reality is socially constructed and subjective. We acknowledge that the experiences and perceptions of mental health in the workplace are shaped by social, cultural, and organizational contexts, and therefore, multiple realities exist based on these diverse experiences.

From an epistemological perspective, we adopt a subjectivist approach. We recognize that knowledge is co-constructed between the researcher and the participants through the research process. This study relies on the rich, detailed accounts provided by participants to generate insights into the detection and disclosure of mental health challenges in the workplace. These elements significantly shape our research approach and findings, as they guide the data collection and analysis processes, ensuring that the voices and perspectives of participants are authentically represented.

### Data collection

Data was collected through a semi-structured interview protocol using purposive sampling method and online information regarding workplace mental health challenges in India was also examined. Semi-structured interviews were conducted with five HR professionals, five counselors, and five employees from five different IT firms with their workplace located in Bangalore, Mumbai, Delhi, Chennai, and Hyderabad – five metropolitan cities in India. We selected IT firms for this study because the IT industry in India is known for its high-pressure environment, long working hours, and demanding nature of work, which significantly impact employees’ mental health and well-being [25]. Semi-structured interviews were chosen as they offer the flexibility to probe deeper into specific areas while maintaining a consistent framework across interviews. This tool enabled us to gather rich, detailed data on personal experiences and professional observations.

The sample included the heads of the HR department, employees who had previously experienced mental health challenge(s), and certified counselors associated with employee assistant programs of respective organizations. All participants were in the age group of 35 to 45 years. Four counsellors identified themselves as female and one as male. Three human resource professionals identified themselves as male and two as female. Three employees identified themselves as female and two as male. All had work experience of more than three years. The sample size was constrained by the point at which the author began to observe repetition in responses. Once the author had interviewed five different respondents from each group, they ceased further interviews, as no new insights were being generated.

The interview protocol was based on the literature on workplace mental health challenges and initiatives undertaken in organizations to combat such challenges [6, 24]. Questions were classified into four groups: one focusing on general information about the participant and his/her association with the organization; second relating to the workplace mental health challenges in their organization; third examining inhibition among employees to share their mental health challenges at the workplace; fourth examining factors that encourage employees to share their mental health challenges and seek-support to combat these challenges; and fifth enquiring into the organizational practices that might help mitigate these challenges at the workplace. The questions were adjusted suitably for interviewing HR professionals, counselors, and employees.

Verbal informed consent was obtained, and confidentiality was assured. Since the topic of workplace mental health is sensitive, all respondents requested non-disclosure of their or their organization’s identity. Participation in the study was completely voluntary. Wherever needed, participants were probed further and were encouraged to provide specific examples to enhance the depth of the data. The interviews averaged 35 min, were recorded in hand-written notes, and transcribed verbatim.

### Data analysis

Data was analyzed using thematic analysis to identify patterns and develop themes. The authors analyzed the data individually and then critically reviewed the identified themes. The authors read the interviews several times to familiarize themselves with the data. Initial codes were developed by identifying recurring patterns, similarities, and dissimilarities across interviews. Then, the codes were critically reviewed to explore commonalities and reconcile differences to develop a common set of initial codes. Next, initial codes were grouped into themes by examining their relationships and interconnections. At this stage, they also examined their themes in light of extant literature on workplace mental health challenges. A final set of themes was developed, enabling them to propose three critical pathways to combat workplace mental health challenges. Table 1 presents the data analysis process and emergent themes during the different phases of data analysis.

**Table 1:** Data analysis process

Phase	Process description	Coders
1. Data familiarization	• Reading interview transcripts multiple times	Authors 1 & 2
2. Initial coding	• Independent coding to identify recurring patterns, similarities, and differences across data • Inter-coder discussion to examine emerging patterns and reconcile differences, if any	Authors 1 & 2
3. Development of initial themes	• Independent coding to group codes into themes, followed by an inter-coder discussion to develop initial themes • Initial themes and sub-themes: • *Inhibition*: Lack of awareness and acceptance, attitude of peers/employer, stigma, fear, low self-confidence, lack of available resources and organizational capability to provide support • *Courage*: Trust, positive relationship at work, perceived social support, communication of examples, confidence in the organization’s capacity to provide support • *Practices*: Creating awareness, providing literacy, strengthening capability, training managers on mental health, leadership advocacy, norms, policies, and processes	Authors 1 & 2
4. Theme consolidation and finalization	• Discussion on themes considering the literature on mental health challenges • Reviewing, refining, and streamlining of themes • Final themes and sub-themes: • *Inhibitory factors*: Lack of awareness, denial, stigma, underestimating organizational capability • *Encouraging factors*: Psychological safety, perceived social support, communicating success stories • *Supportive organizational practices*: Generate awareness and literacy, build organizational capability, strengthen the role of managers, leadership advocacy, policies, and processes	Authors 1 & 2

To enhance the credibility and validity of the findings, triangulation was employed by cross-verifying data from different sources (HR professionals, counselors, and employees). This approach helped in ensuring a more comprehensive understanding of the phenomena under study. By employing thematic analysis, we were able to systematically identify and interpret key themes and pathways related to workplace mental health challenges. This methodological approach provided a robust framework for exploring the nuanced perceptions and experiences of our diverse participant group, contributing to a deeper understanding of the factors influencing detection and disclosure of mental health issues in the workplace.

## Results

**Fig. 1:**
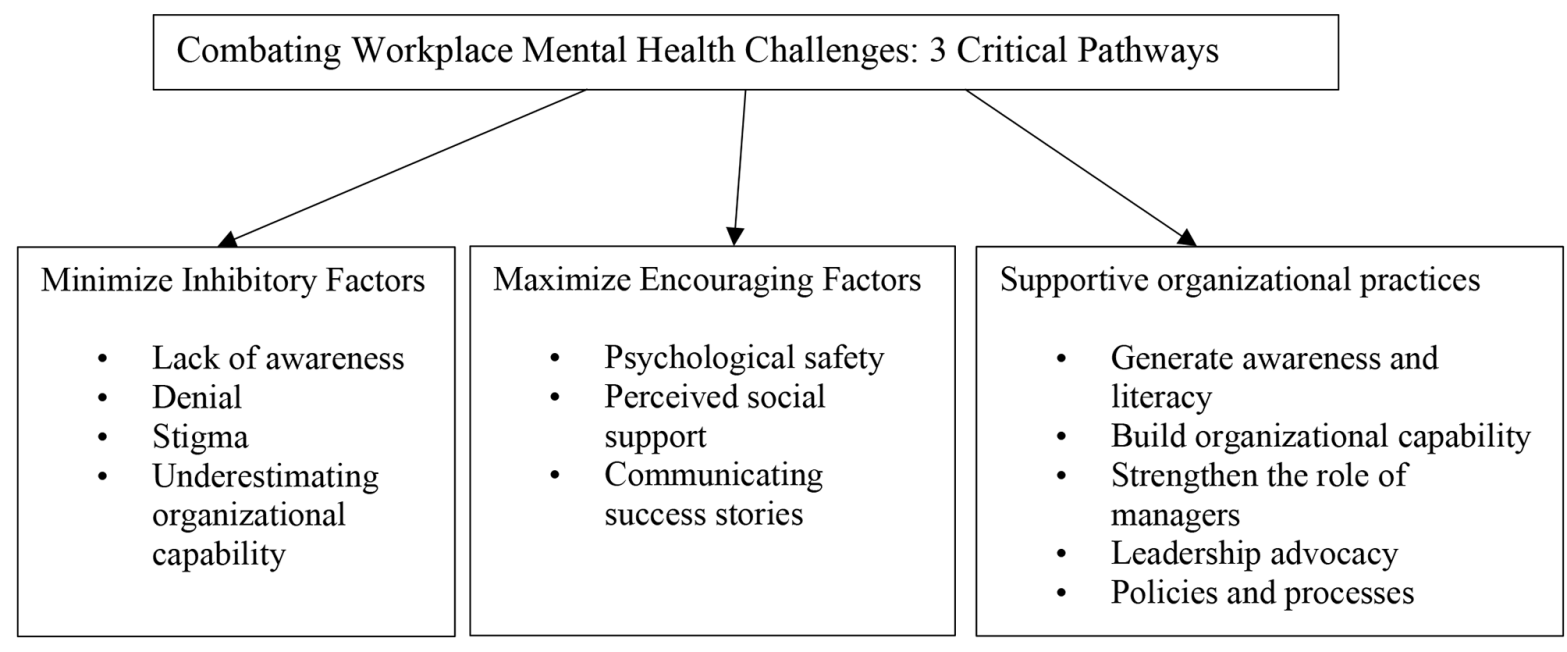
Three critical pathways

Analysis revealed three critical pathways to combat workplace mental health challenges: minimizing inhibitory factors, maximizing encouraging factors, and supportive organizational practices. These pathways are presented in Fig. 1 and are discussed below.

### Minimizing inhibitory factors

To combat workplace mental health challenges, organizations may start by identifying factors that discourage or inhibit employees from disclosing their illnesses. In our interviews with human resource professionals, counselors, and employees, four key factors were identified; these factors, when minimized, tend to promote disclosure and help-seeking behavior among employees at work.

#### Lack of awareness

Although several mental health illnesses are prevalent globally, yet, individuals are uninformed about the causes and symptoms of these illnesses. Specifically, at the workplace, employees do not have the awareness to identify early symptoms of mental health challenges and are unaware of available supportive resources. Multiple counselors advocated this:

There is a lack of understanding and awareness among employees that they are suffering from a mental health challenge. Like, what could be the possible cause(s) that might trigger so and so illness? What is the difference between depression and anxiety, ADHD, or everyday distress?

Even now, there is a lack of awareness around mental health illnesses, and they [employees] are unable to self-identify if these are actual symptoms or something very usual like their day-to-day fatigue.

HR professional further described:

The employee thinks, “I am unsure if this is a mental health issue.” He/she is unaware if the symptoms are related to a mental health illness or something trivial. Most often, they attribute these symptoms to a physical illness, a drop in their energy levels, or a usual low mood.

An employee narrates:

Most of us [colleagues] are unaware of what are the exact symptoms of mental health illnesses. There is a lack of awareness in terms of how illnesses like depression and anxiety manifest in our day-to-day work life. Therefore, it takes a long time before it gets detected, so forget about getting a timely treatment.

Employees even lack awareness about the availability of resources like employee support programs or free online services available *via* their employer. A counselor describes:

Employees are not able to determine whom to approach for these problems within the organization and who would be the right person to discuss their symptoms with. Most of the time, the assumption is that they must reach out for external help or to a close family member.

#### Denial

Employees being in denial of having symptoms of a mental health illness is another inhibitory factor. The lack of acceptance and the non-serious consideration of the early symptoms lead to a denial response. Also, the symptoms of mental health illnesses are not as objectively measured as physical illnesses. Therefore, employees tend to deny or non-accept that they are suffering from mental health illnesses. Also, accepting that one has a mental health illness tends to be traumatic. Therefore, acceptance might become even more difficult.

According to an employee:

I used to feel angry with myself; how could this just happen to me? I could not accept that I was suffering from a mental health problem. It is tough to acknowledge it because you are considered abnormal at work.

HR professional corroborated and stated that denial is one of the inhibitory factors:

Employees do not want to accept that they have mental health challenges. Even if there is a realization that something is wrong psychologically, there is this false belief and overconfidence that they can handle these challenges without any external support.

Employees believe that it is not a big deal to have a mental health challenge and it is just a temporary phase or a mood swing. Many employees have these assumptions and take symptoms of mental health illnesses very lightly, hoping that they will go on their own [without treatment].

The denial response tends to be found more among male employees as they feel an urge to project a tough self-image of themselves at work, and accepting a mental health illness seems to be in opposition to this. Counsellors described:

They feel that boys do [supposed to] not cry. Do I really need assistance for my sadness? Can’t I handle it myself? It becomes an internal battle that they fight with themselves. Accepting or discussing concerns seems to be an act of weakness; therefore, it is not acceptable to them.

It is considered too personal to be shared with anyone, especially in the workplace. Your dirty linen should not be washed in public. If others come to know, it will become a matter of discussion in public space, and I [employee] might be looked down upon.

When an employee was diagnosed with symptoms in a counseling session, he dropped out [dis-continued] to avoid difficult conversations. It is very difficult for him to accept that he needs professional help. It took very long for him to come back for the counselling sessions, and it was sad to see that he was even more depressed.

#### Stigma

The stigma related to mental health illnesses tends to create a sense of fear among employees. They fear that the disclosure of such illnesses might damage their self-identity at work, opportunities would not be given to them, they might not be included as before, and they might even be terminated from their jobs. The coworkers might not be comfortable working with them and tend to judge them as low performers. One of the HR professionals mentioned:

Employees fear adverse impacts on their work in terms of loss of job, poor performance evaluation, no career advancement, and low team inclusion. They might be perceived as underperformers and considered less capable of completing their job responsibilities. There is a fear that other people might learn about it, which will impact their image [self-identity] at work.

Employees are apprehensive to disclose their mental health challenges as they fear that their information will not be kept confidential, damaging their self-identity at work. A sense of embarrassment is also associated with such disclosures. Counsellors describe:

Even after assuring confidentiality, some employees feel I’ll reveal what they shared with me in a session to their HR. This might be further red-flagged to their managers and team members. This is a big fear in them.

She was diagnosed with panic attack disorder and lived in constant fear of being judged at work. She was afraid that it would bring her shame, as it is considered a social stigma even when she was a high performer in her tasks. She feared that peers would label her and that she might be stereotyped.

Employees fear that they will not be included in the decision-making processes and even deprived of further opportunities to grow at work. An employee describes:

When I was diagnosed with adult ADHD, I did not mention it to my manager for several months as I felt he would stop me from meeting clients. It was difficult for me to talk about my difficulties openly as I feared that people might treat me differently and exclude me from the team.

HR professional stated:

Employees feel that managers consider that I am making an excuse for not delivering my work on time. I might be taking advantage of my illness if the manager understands that I have an illness at all. Employees complain that their immediate supervisors or managers sometimes mock them when they discuss our genuine concerns. As HR professionals, when we bring this up with managers, they generally do not show enough trust in employees. They feel employees can use it as an excuse to justify poor work delivery or for falling short on the quality of their work.

#### Underestimating organizational capability

Employees perceive their organization as not yet open and prepared enough to handle workplace mental health challenges. HR professionals describe:

Even with a proper EAP system and in-house counselors, employees think the organization is not equipped or lacks the resources to help them. They believe they will only get some uninformed formal advice, which will be useless.

When I sensed that one of the employees in our organization was extra distressed, I extended help by connecting him to the counselors, but he was so reluctant. He even said, please do not give me gyan [knowledge]; I know how to handle my mind. It took much work to build trust and extend support.

Meanwhile, employees share different perspectives:

You say, “It is okay to be not okay.” However, please let me be not okay even for one day at work. Sending those email posters and having social media campaigns are useless if you cannot even understand my issue.

The access to the counselor is very low. We are a large company with so many employees working here, with access to only a few counselors. I doubt if HR really takes this seriously or if it is just a tick mark on their compliance requirements.

I am unsure if my identity would be kept confidential if I reach a counselor *via* EAP, even though their email says so. It is difficult because you see what happened to the lady who reached out to the counselor earlier this year. You know, everyone came to know about her challenges in our team. I do not trust them enough. Ultimately, all they want is to deliver results, and if we are diagnosed with depression or something, they might rather remove us from the project.

### Maximise encouraging factors

Organizations may also identify factors that encourage employees to disclose their illnesses to combat workplace mental health challenges. In our interviews with human resource professionals, counselors, and employees, three key factors were identified; these factors, when maximized, tend to promote disclosure and help-seeking behavior among employees at work.

#### Psychological safety

When employees feel that their workplace is a safe space to share their mental health illnesses, that their identity would be kept confidential, and that they would still be included in the organization, they tend to disclose their concerns. HR professional describes:

Only when employees believe that it is safe for them to speak up at work, do they talk about their ongoing mental health illnesses. They trust that their information will not be leaked to other members at work, and that they won’t be looked down at.

This lady at my office was confident that we would not take advantage of her sharing about her newly diagnosed mental health challenge and that she is in therapy. We had multiple conversations to support her throughout.

Nowadays, job security is a priority for the employees. They avoid disclosing any information that they think might result in a job loss or termination. So when we make them feel that it is okay to share their concerns and visit the counselors when needed without any implications for their jobs, they seek help.

Counsellors narrate:

Oh, when they come in a session, you won’t believe how many times they tell me, please do not share this with anyone. Only once I assure them of proper confidentiality do they open up and be confident enough to keep coming back.

His manager bullied one of these employees who came to a session with me. He was not ready to talk about him and would avoid conversations related to those incidents. It took a while for me to make him comfortable, build trust, and feel safe to share his ongoing concerns.

Organizations must communicate to the employees that we are appointed to help and support them in distress.

Employees narrate:

When I was undergoing a mental health challenge, my supervisor made me feel safe to speak up with her. Since she genuinely cares for my progress in the organization, she extended help during those difficult days and gave me time to discuss my challenges.

The counselor I was seeing took every step to make me feel safe and heard my concerns without shaming me at all. I think that helped me a lot to share those concerns I had never mentioned to anyone before.

It all boils down to how much you trust your colleagues at work. Sometimes, you are surrounded by people at work who understand you and support you in moving ahead of your dark phases. That was the core of my healing journey.

#### Perceived social support

Employees tend to perceive that if they disclose their mental health challenges, they will be supported due to strong relationships. Their perception matters when reaching out for help and initiating conversations at work. HR professional describes:

It depends on how is the relationship between the employee and his manager. If the bond is based on mutual respect and trust, these challenges will be better handled in the initial stages. Also, the team members play a critical role here.

Empathy is the key, I believe. Anyone can get depressed or anxious at some point in time, and it could be triggered at work. I have to step into the employee’s shoes to understand them.

My doors are open, and I am available for any difficult conversation [around mental health] anytime. Also, if you reach out to me, I am ready to extend any support you might need and connect with you further.

Employee narrates:

Mine wasn’t an easy case, you see. Every time I suffered a panic attack at work, people around me were so concerned and even stopped their work for a minute to get me water or help me calm down. I understand that they might not know exactly what to do to help me, but these little gestures mattered to me. Everyone is busy at work completing their tasks, yet taking a few moments to extend help is a big thing to me.

#### Communicating success stories

In organizations where stories or narratives about how employees could combat their mental health challenges are shared widely, employees were found to be more confident in disclosing and seeking support. An employee narrates:

Is anyone else also reporting their concerns or making an appointment with the counselor? How were they treated when they shared their concerns? What has the organization done to support them? I want to hear the story.

When I watched the video clip by one of my colleagues who could overcome her depression, I felt that she was brave. Later, I saw that she was giving an amazing presentation. It seems like it is normal and could be healed, perhaps. I got hope from her during my difficult days.

HR professional describes:

We share success stories of employees who recovered from depression, anxiety, bipolar, etc., once every month. Most of these stories we pick from the internet. Our own employees are not yet ready to come in front and share. Slowly, awareness is coming post-pandemic, and some of them agree to share their stories.

A counselor narrates:

Organizations should continuously share success stories with their employees. This is important as it gives them confidence. Sometimes, these stories become a precedent for others to follow and come forward to seek help.

### Supportive organizational practices

Organizations may implement a few supportive organizational practices for their employees to combat workplace mental health challenges. In our interviews with human resource professionals, counselors, and employees, five key practices were identified; these practices, when implemented, tend to promote disclosure and help-seeking behavior among employees at work.

#### Generate awareness and literacy

Creating awareness about various mental health illnesses can help employees identify them. Employees could be provided information on common symptoms of stress, depression, anxiety, ADHD, and other mental health illnesses. This would empower the employees to identify and reach out on time. Appropriate corrective measures could follow this. A counselor narrates:

Organizations must educate employees about various mental health challenges. These are curable and shall be reported promptly. Common symptoms of these psychological problems should be clearly explained to employees so that they can self-identify them.

HR professional describes:

We have included an awareness session on mental health during the employee induction program. This session introduces common mental health challenges that an employee might face at work and what resources are available to them. Whom to reach out to and the process to follow in such incidences are clearly explained to them.

Recently, we invited a psychiatric doctor who conducted an interactive workshop on how to identify symptoms of the most common mental health illnesses. We regularly circulate different posters, videos, and content on this with our employees.

#### Build organizational capability

Organizations shall take measures to build resources to manage mental health challenges at work. Employees do assess their organization’s readiness to accept and support them if they disclose their mental health challenges. A counselor describes:

Organizations must create a safe space through anonymous channels such as dedicated email addresses or helpline numbers for employees to seek support. These channels should be trustworthy and, in most cases, handled by external or senior managers of the organization.

A counselor narrates:

Employers shall provide their employees with access to counseling services. Investing in employee assistance program (EAP) services is helpful. Also, appointing an in-house counselor full-time or even part-time helps to handle a large number of employee cases.

While providing such services as a part of organizational compliance is a mandate, constant communication about how to access these services is also essential. An HR professional narrates:

We have ERP in place, but for employees to actually benefit from these services, we keep sending them updates. Our counseling agency also circulates newsletters on mental health. We conduct awareness campaigns on social media, online learning programs are available, and sometimes we also invite mental health specialists to lead workshops.

#### Strengthening the role of managers

Managers tend to play a key role in identifying employees who might be showing symptoms or suffering from mental health illnesses. Their behavior either supports or discourages an employee from disclosing their concern. An HR professional narrates:

On one hand, managers are the first to recognize symptoms and let us know; on the other hand, they are the ones who make it difficult for the employee to speak up. They have to get the work done by the employee, so sometimes they think he/she is making an excuse to avoid responsibility. We do see trust issues in these cases. After seeing several such cases, we have also started to train and empower our managers. Their understanding might help employees to seek support and get timely assistance.

Managers are critical in checking such deviations and reporting such cases to us. Meanwhile, we also assure the confidentiality that adverse consequences won’t happen to them if they let us know about someone in need.

A counselor describes:

Managers must be trained in emotional intelligence, which will help them to respond with empathy. If they sense a behavior change, they may offer help or connect them with us as they work together.

Employees describe:

If you wish to make any dent in mental health at work, please train our managers. They project as if they understand, but I seriously doubt they know anything about mental health challenges and how to deal with someone who might be going through one.

My manager was my go-to person; she listened to my concerns and gave me time. She never mocked me or made me feel excluded on work projects. If she had not given me the space, I could not have voiced myself.

#### Leadership advocacy

Leaders in the organization also contribute significantly to making workplaces ready and equipped to deal with mental health challenges. Their genuine intention to invest in resources to extend support to their employees is beneficial. Some leaders are open about sharing their own experiences and build confidence in their employees to voice their concerns. An HR professional narrates:

When leaders give priority to handling mental health challenges, they invest time and build resources for the employees. They talk about such challenges openly in multiple forums. They also mention how critical mental health is for employee performance. Some even share their own stories about what helped them overcome those challenges.

Our leaders emphasize the robust mental health and well-being of employees. They invest money in conducting workshops and implementing practices and policies to support any employee in need.

A counselor narrates:

Leaders must demonstrate vulnerability and self-exposure. They must share experiences from their real life, which will make employees feel comfortable talking about their challenges.

#### Policies and processes

Clearly defined policies and fair processes to address mental health challenges are a growing concern. Implementation of these policies is also essential. HR professionals describe:

We are building a culture of inclusion and taking steps to implement it. We are also constantly upgrading our policy document to protect our employees. We intend to communicate the process to all members and their responsibilities in each step. Our goal is to have a fair process for all employees within the company.

Policies for mental health are still in their infancy at my company. A lot more education, awareness, and intention would be needed to actually get ready to address the growing number of mental health cases we see now.

Regular check-ins with employees via in-house surveys are what we have initiated. However, we need to do more.

Mental health challenges impact employee performance, but discriminating against them on this basis might be wrong. A more precise policy might protect them.

A counselor suggests:

Policies that cover mental illness as any other physical illness are essential. Employees complain about the high counselors’ fees or that therapy is expensive. Making these services accessible by employers or on subsidiary rates could be helpful.

From induction itself, employees must be notified of available resources. How do they raise concerns? What will be the process to apply for leave if they are diagnosed with any mental health disorder and are advised to rest?

Employees narrate:

I think we are still growing up in terms of policies to protect us. Management has to look into this seriously. Many employees find it difficult to report or discuss their concerns because they don’t trust the process or fear layoff, as we are not covered via any policy.

## Discussion

This exploratory study examines the critical pathways of combating workplace mental health challenges in the Indian context. Since mental health challenges at the workplace are a growing concern in Indian organizations that adversely impact the productivity of employees, timely detection of these challenges is very important. Though many employees experience these mental health challenges, most do not disclose and seek support due to multiple reasons, including a lack of psychological safety and stigma associated with the same. We examined these inhibitory factors, encouraging factors, and supportive organizational practices that tend to impact the detection and disclosure of workplace mental health challenges among employees. This has implications for theory and practice.

### Inhibitory factors

In Indian workplaces, one of the primary inhibitory factors identified is the pervasive lack of awareness and understanding about mental health issues [7]. Many employees and even managers may not recognize the signs and symptoms of mental health challenges, leading to underreporting and inadequate support [12, 35, 49]. Moreover, cultural stigmas associated with mental illness play a significant role. In India, discussing mental health problems openly is often seen as a sign of weakness or instability, which discourages individuals from seeking help [36, 37].

Fear of stigma is another critical inhibitory factor. Employees fear being judged or discriminated against by colleagues and supervisors if they disclose mental health issues. This fear is compounded by concerns about job security, as there is a prevalent belief that admitting to mental health challenges could lead to discrimination or even dismissal [37–39]. Confidentiality concerns also feature prominently. Employees worry about the confidentiality of their disclosures and whether their personal information will be handled sensitively by the organization. This uncertainty further deters individuals from seeking the necessary support they may require [10, 12, 14].

### Encouraging factors

Despite these challenges, several factors can encourage employees to disclose and seek help for mental health issues. Creating a psychologically safe environment is crucial. When organizations foster an atmosphere where employees feel safe to discuss their mental health concerns without fear of repercussions, individuals are more likely to seek assistance [36, 40]. Promoting social support within the workplace also plays a significant role in encouraging disclosure. When employees perceive that they have the support of their colleagues and supervisors, they are more inclined to open up about their mental health challenges and seek help [15, 41, 42].

Open communication about mental health issues is another encouraging factor. Normalizing conversations around mental health and providing forums where employees can discuss their experiences openly help reduce stigma and encourage early intervention [42, 43]. Sharing success stories of employees who have managed mental health challenges effectively can serve as powerful motivators. These stories demonstrate that seeking help is not only acceptable but also beneficial for personal well-being and career growth.

### Supportive practices

Our study highlights several supportive organizational practices that can enhance mental health support in Indian workplaces. Implementing comprehensive training programs to increase mental health literacy among employees and managers is essential. These programs educate staff about common mental health issues, how to recognize symptoms, and where to seek help [36, 44, 45]. Strengthening counseling services and offering accessible mental health resources, such as online workshops and peer support groups, can mitigate barriers to seeking help [8, 46, 47]. Empowering supervisors to play an active role in supporting employees’ mental health and fostering trusting relationships within teams are crucial steps toward creating a supportive organizational culture [41, 48].

### Practical implications and recommendations

The socio-cultural context of India plays a pivotal role in shaping attitudes toward mental health and help-seeking behaviors [49]. Cultural beliefs, societal norms, and the pervasive stigma surrounding mental health significantly impact both the detection and disclosure of mental health issues in the workplace [50]. Our findings suggest that in India, there is a strong stigma attached to mental health issues, which often leads to fear of judgment, discrimination, and professional repercussions. Employees are reluctant to disclose their mental health challenges due to the fear of being perceived as weak or incapable. This stigma is compounded by a lack of awareness and understanding of mental health symptoms and illnesses, causing many employees to be in denial about their mental health issues. Addressing this stigma through organizational policies and culture is crucial for encouraging help-seeking behaviors. Organizations must actively work towards destigmatizing mental health issues through awareness campaigns, open discussions, and integrating mental health into their wellness programs [37, 49, 51]. This can help reduce fear and encourage employees to seek help.

Also findings indicate that there is a significant gap in awareness regarding mental health symptoms and the availability of support resources. Many employees do not recognize the signs of mental health challenges or are unaware of the support systems within their organizations. Enhancing mental health literacy through education can play a crucial role in facilitating help-seeking behaviors. By improving awareness and understanding, organizations can reduce stigma and encourage early detection and disclosure of mental health issues [52]. Conducting regular mental health education and training sessions can improve mental health literacy among employees, helping them recognize symptoms early and understand the importance of seeking professional help [34, 38].

Indian workplaces often have hierarchical structures, where power distance and authority can influence an employee’s willingness to seek help. Our findings indicate that the fear of losing respect or job security can deter employees from disclosing their mental health issues. Training managers to recognize mental health issues and provide support can create a more supportive atmosphere for employees [37]. Managers play a critical role in shaping the work environment, and their involvement is essential for fostering a culture of openness and support. Providing training for managers to identify and support employees with mental health challenges can create a more empathetic and supportive work environment [15, 16]. Managers should be equipped with the skills to handle such issues sensitively and effectively.

Implementing policies that foster psychological safety, confidentiality, and inclusivity can encourage employees to seek help without fear of repercussions. Organizations need to build trust and ensure that employees feel safe to disclose their mental health concerns. Ensuring confidentiality and creating a culture of trust where employees feel safe to disclose their mental health concerns without fear of negative consequences is essential [16, 40]. Organizations should establish clear policies and practices to protect employee confidentiality.

Leadership advocacy is also crucial; when leaders prioritize mental health and openly discuss it, it sets a positive precedent for the entire organization. Creating a supportive culture where mental health is integrated into wellness programs can significantly impact help-seeking behaviors [33]. Leadership must actively promote mental health initiatives and demonstrate their commitment to employee well-being. This includes advocating for mental health resources and being transparent about the organization’s mental health policies and support systems.

Employees need to believe that their organization is capable of providing effective mental health support. If they perceive the organization as ill-equipped or insincere in its efforts, they are less likely to seek help. Organizations must demonstrate their commitment to mental health through tangible actions and resources, ensuring that employees have confidence in the support systems available to them [7, 49]. By considering these socio-cultural factors and implementing supportive organizational practices, Indian workplaces can better facilitate mental health-related help-seeking, ultimately fostering a healthier and more resilient workforce.

### Critical reflexive understanding

As researchers, we recognize that our own perspectives and backgrounds have influenced this study. Conducting research in a culturally diverse and rapidly developing economy like India presents unique challenges and opportunities. Our assumptions about mental health, shaped by both global and local discourses, have inevitably influenced our interpretation of the data. Throughout the research process, we remained conscious of our positionality and worked iteratively between exsisting literature and data. The decision to focus on semi-structured interviews allowed us to capture nuanced insights, but it also required us to navigate sensitive conversations with empathy and cultural sensitivity. We acknowledge that our presence as researchers might have influenced participants’ responses, particularly in a context where discussing mental health openly is still stigmatized. Also our backgrounds in organizational psychology and human resource management provided us with a particular lens through which to analyze the data. This has both strengths and limitations. While it enabled us to deeply understand organizational dynamics, it also meant that we had to be vigilant about not imposing our own frameworks onto participants’ experiences. To mitigate this, we engaged in regular reflexive discussions among us and included diverse perspectives in our thematic analysis [53].

By integrating critical reflexive understanding into the discussion, we hope to provide a more nuanced and comprehensive analysis of workplace mental health challenges in India. We acknowledge, that the limited number of participants could impact the generalizability of our findings. However, these narratives provided valuable insights into the lived experiences of employees, counselors, and HR heads, highlighting the complexities of mental health challenges in the workplace. Our findings underscore the importance of culturally sensitive approaches and the need for organizations to actively foster supportive environments. Future research should continue to explore these themes, with a particular focus on longitudinal studies to assess the long-term impact of organizational practices on mental health outcomes.

### Electronic supplementary material

Below is the link to the electronic supplementary material.

Supplementary Material 1

## Data Availability

Data is provided as a supplementary file.
